# Dissimilarity measure of local structure in inorganic crystals using Wasserstein distance to search for novel phosphors

**DOI:** 10.1080/14686996.2021.1899555

**Published:** 2021-04-21

**Authors:** Shota Takemura, Takashi Takeda, Takayuki Nakanishi, Yukinori Koyama, Hidekazu Ikeno, Naoto Hirosaki

**Affiliations:** a Sialon Group, National Institute for Materials Science, Tsukuba, Japan; b Research and Services Division of Materials Data and Integrated System (MaDIS), National Institute for Materials Science, Tsukuba, Japan; c Department of Materials Science, Graduate School of Engineering, Osaka Prefecture University, Sakai, Japan

**Keywords:** Phosphors, local structure, similarity measure, Wasserstein distance, 40 Optical, magnetic and electronic device materials

## Abstract

To efficiently search for novel phosphors, we propose a dissimilarity measure of local structure using the Wasserstein distance. This simple and versatile method provides the quantitative dissimilarity of a local structure around a center ion. To calculate the Wasserstein distance, the local structures in crystals are numerically represented as a bag of interatomic distances. The Wasserstein distance is calculated for various ideal structures and local structures in known phosphors. The variation of the Wasserstein distance corresponds to the structural variation of the local structures, and the Wasserstein distance can quantitatively explain the dissimilarity of the local structures. The correlation between the Wasserstein distance and the full width at half maximum suggests that candidates for novel narrow-band phosphors can be identified by crystal structures that include local structures with small Wasserstein distances to local structures of known narrow-band phosphors. The quantitative dissimilarity using the Wasserstein distance is useful in the search of novel phosphors and expected to be applied in materials searches in other fields in which local structures play an important role.

## Graphical abstract

**Figure:**
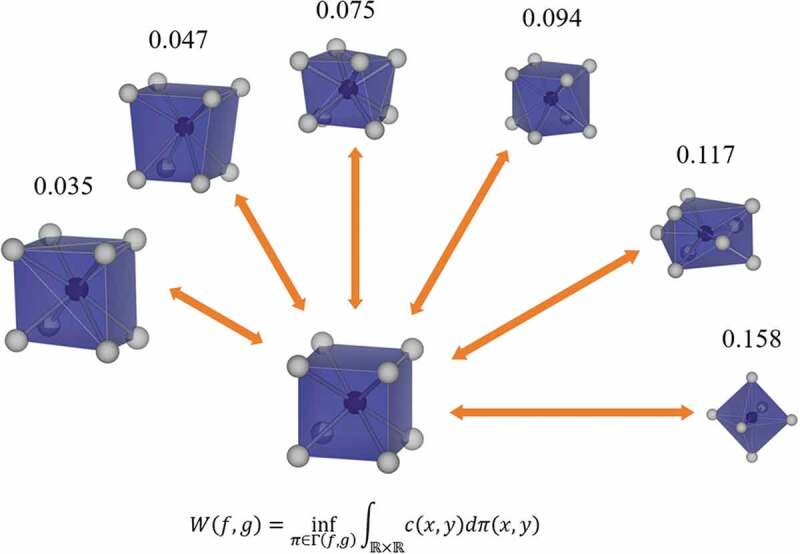

## 1. Introduction

Phosphors are used for general lighting and display backlight. Transition metal ions and rare earth ions are used as an emitting ion for phosphors. The emitting color of the phosphors depends on the luminescence properties such as the peak wavelength and the emission spectral width. To realize a novel phosphor, the luminescence properties must be controlled. The emissions of Ce^3+^ and Eu^2+^ based on a 4 f-5d transition can be controlled by changing the local structure around the ions. An 8 K TV requires next-generation green and red Eu^2+^ phosphors with a narrow-band emission to satisfy BT.2020 standard. Eu^2+^ phosphors with a narrow-band emission are reported for CaF_2_ [1], BaSi_2_O_2_N_2_ [2], CaAl_2_S_4_ [3], β-Sialon [4–6], etc. The full width at half maximum (FWHM) of the emission spectrum also depends on the local structure in the phosphor. However, the ideal local structure with the narrowest FWHM is unknown theoretically. Since Eu^2+^ in CaF_2_, which is a simple crystal with a cubic local structure, and UCr_4_C_4_-type phosphors with a cubic-like local structure such as SrLiAl_3_N_4_:Eu^2+^ [7], SrMg_3_SiN_4_:Eu^2+^ [8], CaLiAl_3_N_4_:Eu^2+^ [9] and RbLi(Li_3_SiO_4_)_2_:Eu^2+^ [10] show a narrow-band emission, a crystal structure with a cubic local structure has been garnering attention as a novel narrow-band phosphor candidate.

Currently, an efficient approach by material informatics has been used to discover candidate materials from a large amount of data. This method has been applied to search for novel phosphors and interpret relationship between the luminescence properties and material attributes [11–13]. Kim et al. extracted K_2_Zn_6_O_7_, which has a cubic-like local structure, and 37 other kinds of crystal structures by screening the crystal structures in the Inorganic Crystal Structure Database (ICSD) under various conditions [13]. In addition, they developed a Sr_2_MgAl_5_N_7_:Eu^2+^ narrow-band phosphor as an element substitute from K_2_Zn_6_O_7_. Zimmermann et al. reported a method to determine the coordination number of a local structure [14], and expressed the local structure as a vector by defining the local structure order parameters [14–16]. Zimmerman’s method can classify local structures.

Another method to efficiently screen for a new phosphor is to search for a similar local structure to that in a known practical phosphor. Kim et al.’s method is qualitative because the similarities of the extracted cubic-like structure to the cubic one are not listed. Therefore, quantification of the similarity between the basis local structure and obtained local structure is required. Quantification can directly search for a host crystal, which includes the objective local structure. On the other hand, Zimmermann et al.’s procedure is complex because a new coordinate system must be set to the local structure and many parameters can be introduced. Consequently, a simpler method to obtain the similarity is required. Moreover, a cubic structure is not the only that shows a narrow-band emission.

Therefore, a simple and versatile method to obtain quantitative similarity for a local structure around the center ion is necessary to develop a more efficient phosphor search. In this work, we define the local structure in an inorganic crystal as a geometrical one consisting of the center ion and the surrounding ligands. The local structure is represented as a distribution of the interatomic distances, which consist of the center-ligand distances and ligand-ligand distances. Since the local structure is represented as a distribution, this approach can be applied to any local structures from cube with high symmetry to highly distorted local structures with C_1_ symmetry. By expressing the local structure as a distribution, the difference in distribution can be obtained by the method of data science. There are some indicators which showing the difference in distribution. In this study, we propose a dissimilarity measure using the Wasserstein distance. The Wasserstein distance is typically used for image and audio processing as well as generative adversarial networks [17–19]. The Wasserstein distance, which is based on the transport problem, is the distance between the distributions, and the calculation does not require any parameters. In the field of data science, a (dis)similarity between data corresponds to a distance between data. Therefore, we can obtain and quantify the dissimilarity between local structures by the Wasserstein distance. Transport problem is calculation of the minimum cost for transporting luggage from one point to another point, and thus the obtained Wasserstein distance in this study is the minimum deformation for changing a local structure that is a polyhedron to another local structure. Hence, a small value of the Wasserstein distance indicates that the local structures are similar, while a large value implies that they are dissimilar

To demonstrate the efficiency in searching for novel phosphors, we performed a calculation of the Wasserstein distance to obtain the quantitative dissimilarity between various local structures. Specifically, the Wasserstein distances were calculated in three cases. First, we calculated the difference between characteristic ideal structures such as cubic and confirmed that the variation of the Wasserstein distance corresponds to the structural variation of the local structures. Second, we calculated the difference between the ideal structure and the local structure in known phosphors, demonstrating that the quantitative dissimilarity is effective for actual structures. Finally, we calculated 170 local structures in a known Eu^2+^ phosphors [3,10,20–24], the cubic-like Sr1 site in SrLiAl_3_N_4_, and the 9-coordinated structures in β-Sialon, which are narrow-band phosphors. Then, we discussed the relations of the Wasserstein distance and FWHM of the phosphors. The results confirm that the quantitative dissimilarity by the Wasserstein distance is useful to search for novel phosphors. This method should also be applicable to materials searches in other fields where the local structures in crystals play an important role.

## 2. Computational procedure

### 2.1. Local structure representation

In this work, the local structure in the crystal is defined as a geometrical structure consisting of the center ion and the ligands. The numeric representation of the local structure is required to be invariant to a translation, rotation, and exchange of the order of the ligands. Accordingly, we propose that the local structure is represented as a bag consisting of the center-ligand and ligand-ligand distances. The bag is expedient and an invariant to a translation, rotation, and exchange of the order of the ligands. However, it lacks the correspondence between atoms. A bag of interatomic distances of an *N*-coordinated ion is therefore represented by a bag of *N* center-ligand distances and *N*(*N*-1)/2 ligand-ligand distances, namely,

(1) $$D=\left\{d_{1}^{CL},\cdots ,d_{N}^{CL},d_{1,2}^{LL},d_{1,3}^{LL},\cdots ,d_{N-1,N}^{LL}\right\},$$

where $d_{i}^{CL}$ and $d_{i,j}^{LL}$ are distances between the center ion and *i*-th ligand, and between *i*-th and *j*-th ligands, respectively. The distances can be normalized by the average center-ligand distance, $\bar{d^{CL}}$, to cancel the difference in ionic radii of the center ions, as

(2) $$\tilde{D}=\left\{d_{1}^{CL}/\bar{d^{CL}},\cdots ,d_{N}^{CL}/\bar{d^{CL}},d_{1,2}^{LL}/\bar{d^{CL}},\cdots ,d_{N-1,N}^{LL}/\bar{d^{CL}}\right\},$$

(3) $$\bar{d^{CL}}=\frac{1}{N}\overset{N}{\underset{i=1}{\sum }}d_{i}^{CL},$$

The bag representation of the normalized distances is used in this study. Since the coordination number must be determined to extract the local structure from the actual crystal structure, we employed the CrystalNN method [15]. Here, the bonds are assumed to be equivalent but other cases can be assumed. For example, the ratio of the ligand-ligand distances in the bag increases when the coordination number increases because the number of ligand-ligand distances is $N\left(N-1\right)/2$ where N is the coordination number. It can be corrected by assigning different weights to the center-ligand and the ligand-ligand distances.

### 2.2. Dissimilarity measure of local structures

The distance bag can be expressed as the distribution of the interatomic distances, which is given as

(4) $$f(x;\tilde{D})=\frac{1}{\left|\tilde{D}\right|}\underset{d\in \tilde{D}}{\sum }\delta \left(x-d\right),$$

where $\left|\tilde{D}\right|$ is the number of elements in the bag $\tilde{D}$, and $\delta \left(.\right)$ is the Dirac delta function. In this study, we propose that the dissimilarity of local structures can be defined as the Wasserstein distance, which is the distance between the distance distributions. The Wasserstein distance between one-dimensional distributions of *f* and *g* is expressed as

(5) $$W\left(f,g\right)=\underset{\pi \in \Gamma \left(f,g\right)}{\inf}\int_{R\times R}^{\;}c\left(x,y\right)d\pi \left(x,y\right),$$

where $c\left(x,y\right)=\left|x-y\right|$ is the cost matrix of the transport distances, and $\Gamma \left(f,g\right)$ *is a set of all couplings of f* and *g* that have marginals *f* and *g* on the first and second factors, respectively. The Wasserstein distance is calculated using SciPy packages [25]. If two local structures are congruent or geometrically similar in terms of geometry, *W* is 0.

**Figure 1.**
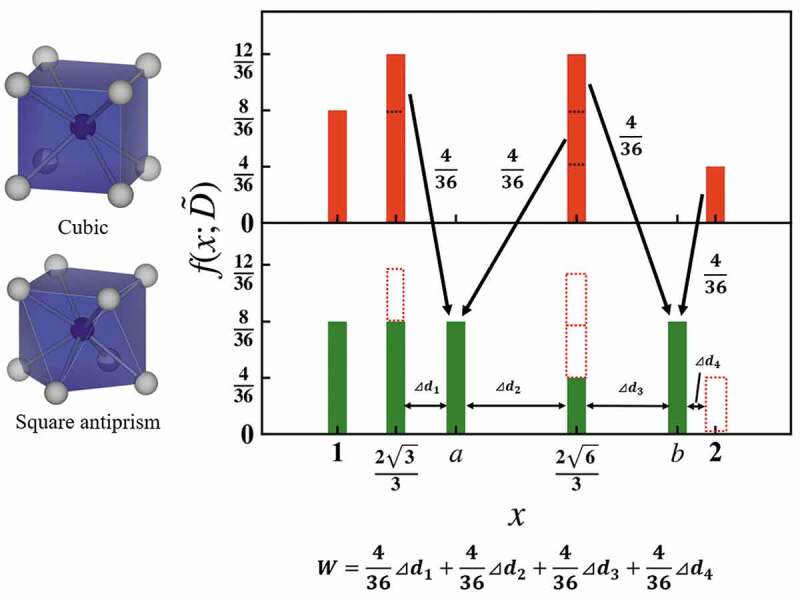
Scheme of Wasserstein distance between a cubic and a square antiprism that distorts the helix angle 45 degrees from cubic. TSTA_A_1899555 is the transport distance. a and b are $\frac{\sqrt{3\left(1+2cos^{2}\frac{3}{8}\pi \right)}}{3}$ and $\frac{\sqrt{3\left(3-2cos^{2}\frac{3}{8}\pi \right)}}{3}$, respectively

Figure 1 schematically shows the calculation of the Wasserstein distance between a cubic and a square antiprism that distorts the helix angle 45 degrees from cubic keeping its height as an example. When a cubic structure is expressed as the bag in Equation (1), the bag consists of eight center-ligand distances, which are normalized to 1 and the 28 normalized ligand-ligand distances. The normalized ligand-ligand distances are twelve $\frac{2\sqrt{3}}{3}$, twelve $\frac{2\sqrt{6}}{3}$, and four 2. In the case of square antiprism, the eight center-ligand distances are the same for cubic, except 28 ligand-ligand distances differ from the cubic one. The ligand-ligand distances are eight $\frac{2\sqrt{3}}{3}$, eight $\frac{\sqrt{3\left(1+2cos^{2}\frac{3}{8}\pi \right)}}{3}$, four $\frac{2\sqrt{6}}{3}$, and eight $\frac{\sqrt{3\left(3-2cos^{2}\frac{3}{8}\pi \right)}}{3}$. The distributions of cubic and square antiprism in Equation (2) are indicated in Figure 1 as a histogram, where the arrows denote the optimal transport and the numbers next to the arrows are the transport cost. W is calculated as the sum of the products of the transport distance and the transport cost. In this case, W is 0.094.

## 3. Results and discussion

### 3.1. W between ideal structures

**Figure 2.**
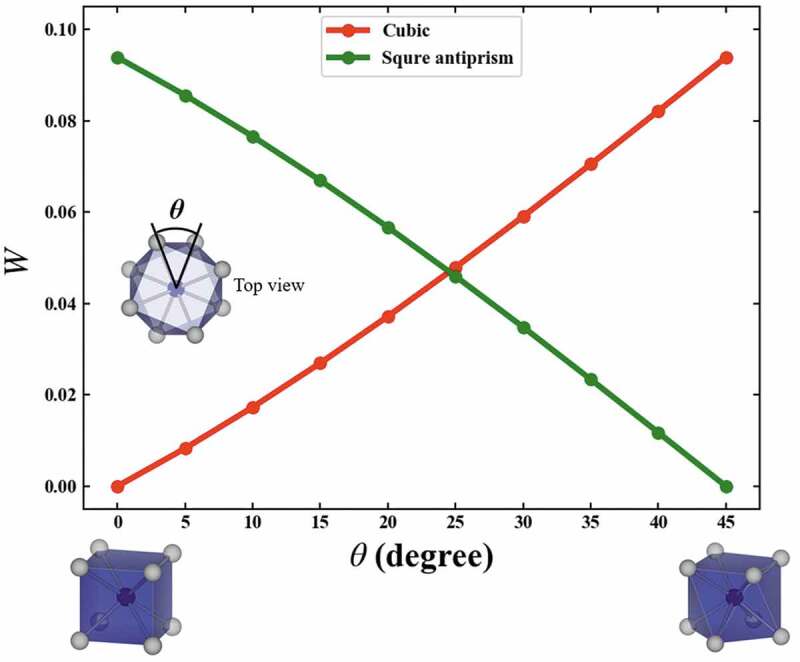
Variations of the W which correspond to the structural variation of the helix angle θ from a cubic to a square antiprism. Red line indicates the W between cubic and the structure with θ, while the green line indicates the W between square antiprism with θ = 45 and the structure with θ

**Figure 3.**
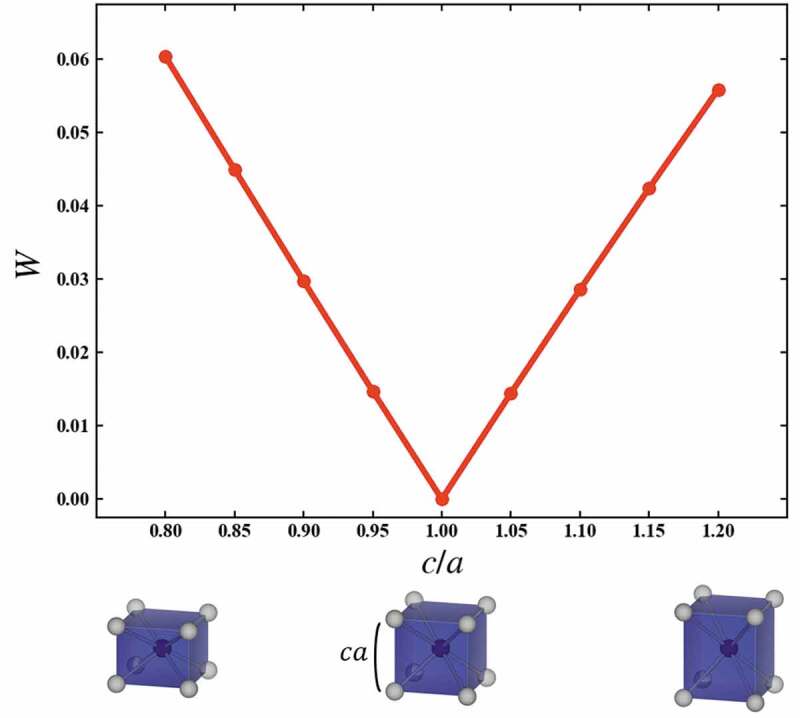
Variations of the *W*, which correspond to the structural variation of the *c*/*a* from cubic to rectangular parallelepiped

Figure 2 shows the variations of *W* corresponding to the structural variation of the helix angle *θ* from a cubic to a square antiprism. The red line indicates *W* between cubic and the structure with *θ*, while the green line indicates *W* between square antiprism with *θ *= 45° and the structure with the *θ. W* is calculated in units of 5 degrees for helix angle *θ*. The variations of *W* are monotonic. Since *W*’s to cubic and square antiprism cross at 0.047. The cubic is changed by one-dimensional distortion to a flat and slender rectangular parallelepiped. The variations of *W* which correspond to the structural variation of *c*/*a*, where *a* and *c* are respectively width and height of the rectangular parallelepiped, from a cubic to a rectangular parallelepiped are shown in Figure 3. The variations of *W* are also monotonic. From these results, the variation of the *W* corresponds to the structural variation of the local structures, and *W* can quantitatively explain the similarity between local structures.

In this approach, *W* can be calculated as the local structures with different coordination numbers because the local structures are expressed as the distance distribution. To calculate *W* of the local structures with the different coordination numbers, seven kinds of ideal structures were selected: tetrahedron, trigonal bipyramid, octahedron, pentagonal bipyramid, cubic, square antiprism, and cuboctahedron. The center-ligand distances are the same in each ideal structure. Table 1 shows *W* of the seven kinds of ideal structures. The *W* between pentagonal bipyramid and square antiprism is the smallest at 0.067, but pentagonal bipyramid and square antiprism are dissimilar because the value is larger than 0.047 in Figure 2. Local structures with different coordination numbers indicate dissimilarity. Therefore, this quantitative dissimilarity can be calculated for any local structure, and a local structure with a different coordination number is dissimilar spontaneously.

**Table 1.** *W* among the various structures with different coordination numbers

	Tetrahedron	Trigonal bipyramid	Octahedron	Pentagonal bipyramid	Cubic	Square antiprism	Cuboctahedron
							
Coordination number	4	5	6	7	8	8	12
Tetrahedron	0	0.145	0.200	0.179	0.143	0.179	0.131
Trigonal bipyramid	0.145	0	0.080	0.082	0.141	0.120	0.093
Octahedron	0.200	0.080	0	0.086	0.158	0.151	0.167
Pentagonal bipyramid	0.179	0.082	0.086	0	0.127	0.067	0.127
Cubic	0.143	0.141	0.158	0.127	0	0.094	0.111
Square antiprism	0.179	0.120	0.151	0.067	0.094	0	0.120
Cuboctahedron	0.131	0.093	0.167	0.127	0.111	0.120	0

### 3.2. W between the ideal structure and the actual local structure in known phosphors

**Figure 4.**
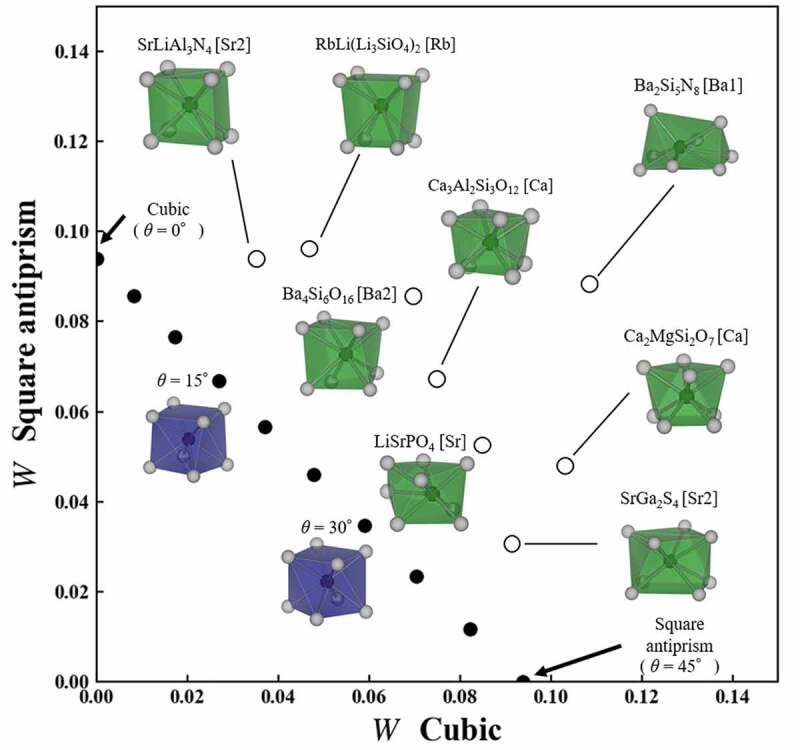
Diagram of the *W* to cubic and square antiprism of eight local structures of the known phosphors

The 170 local structures were extracted from crystal structures registered in ICSD, except for solid solutions, partial occupancy. The central ion of the local structure was an alkaline metal, alkaline earth metal, or rare-earth ion. Figure 4 shows *W* to cubic and square antiprism of eight local structures, which are selected from the 170 structures of known phosphors. The x and y axes indicate *W* to cubic and *W* to square antiprism, respectively. Cubic is located at (0,0.094) on the y axis, while square antiprism is located at (0.094,0) on the x axis. Black circles in a diagram indicate *W* of square antiprisms with helix angle *θ* to cubic and square antiprism. When the local structure has a distortion different from *θ*, the coordinate recedes from the points of the square antiprism structure to the upper right. In this paper, to distinguish the local structure in the crystal structure, we use a notation with a central ion after the chemical formula. For example, the Ca site of CaF_2_ is expressed as CaF_2_[Ca]. When there are multiple sites of the same ion in the crystal structure such as SrLiAl_3_N_4_, the Sr1 and Sr2 sites are expressed as SrLiAl_3_N_4_[Sr1] and SrLiAl_3_N_4_[Sr2], respectively. SrLiAl_3_N_4_[Sr2], which is the furthest on the left in the diagram, is most similar to cubic in the eight structures. On the other hand, SrGa_2_S_4_[Sr2] at the bottom in the diagram is most similar to square antiprism in the eight structures. Ba_2_Si_5_N_8_[Ba1] at the top right is dissimilar to both cubic and square antiprism. The dissimilarity using Wasserstein distance is accurately quantified in the actual local structures.

### 3.3. W between actual local structures in known phosphors

**Figure 5.**
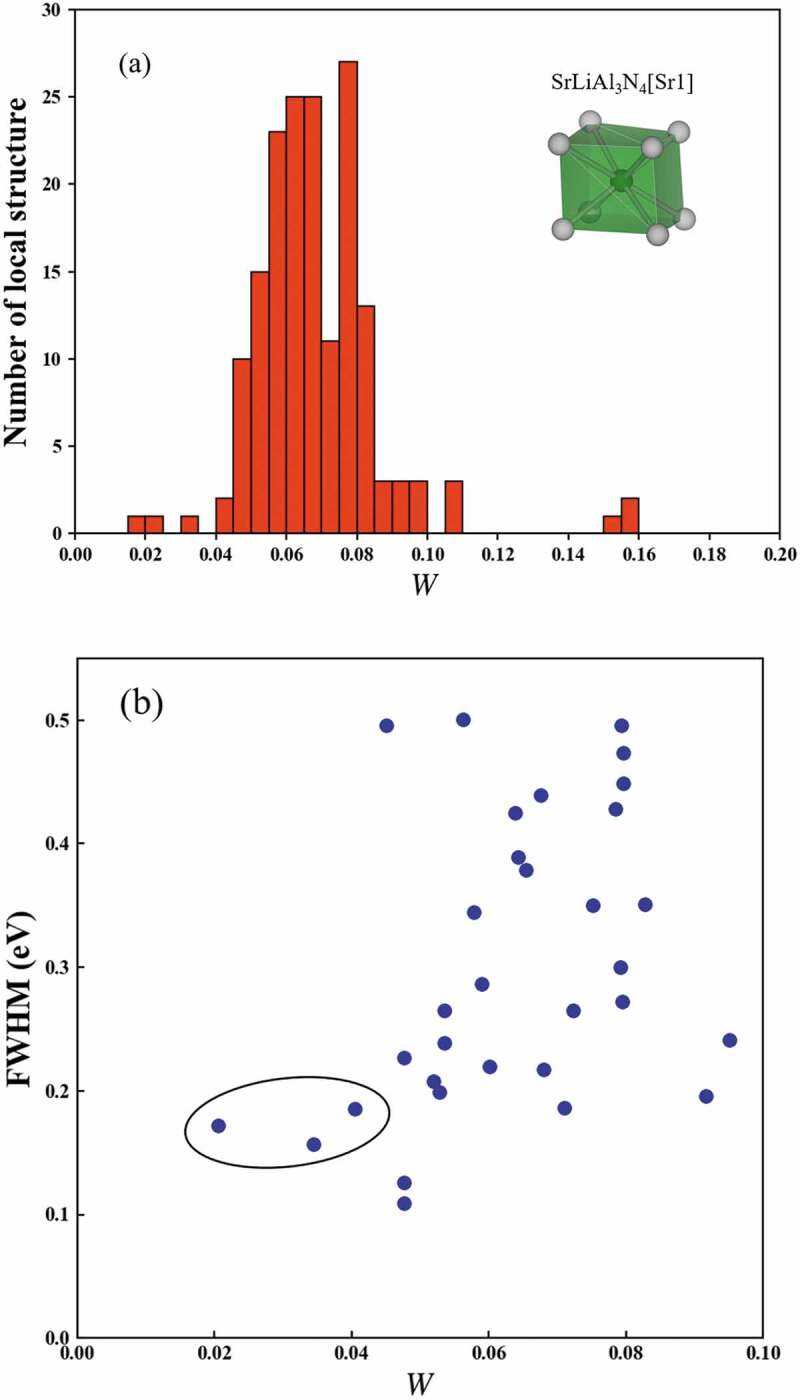
(a) Distributions of the *W* to SrLiAl_3_N_4_[Sr1] of 169 local structures and (b) correlation of *W* to SrLiAl_3_N_4_[Sr1] and the FWHM of 32 local structures of the single Eu substituted sites

Figure 5(a) shows the distribution of *W* to the SrLiAl_3_N_4_[Sr1]. *W* of most of local structures are distributed between 0.045 and 0.100. However, there are a few phosphors with a small *W* in 0–0.045. Figure 5(b) shows the correlation of *W* to SrLiAl_3_N_4_[Sr1] and the FWHM of the local structures. For brevity of the discussion, only 32 local structures of the single Eu substituted sites in the phosphors are illustrated. Although multiple sites can be substituted in Eu^2+^ phosphors, the discussion between the FWHM of the emission spectrum and the local structure is difficult due to effects such as inhomogeneous broadening. In Figure 5(b), there are three local structures with a small *W* surrounded by black circle. Those phosphors are the UCr_4_C_4_-type phosphors with a narrow-band emission. On the other hand, there are many local structures with *W* more than 0.045, and those values of the FWHM are scattered.

From these results, if *W* of the structures is small, the values of the FWHM are similar. In contrast, if *W* is large, the correlation of the FWHM is fading. There are various local structures that are dissimilar to SrLiAl_3_N_4_[Sr1]. Some local structures result in narrow band emissions and others in broad band emissions. It is not obtained from W how local structures are formed. Further research is necessary in the future. SrLiAl_3_N_4_:Eu^2+^ shows a narrow band emission despite having two substitution sites in the crystal structure. *W* between SrLiAl_3_N_4_[Sr1] and SrLiAl_3_N_4_[Sr2] is 0.016, which is the minimum in *W* in the 169 local structures. This value indicates the two Sr site in SrLiAl_3_N_4_ are very similar. Accordingly, the narrow-band emission of SrLiAl_3_N_4_:Eu^2+^ is due to the restraint of inhomogeneous broadening by the similarity of two Sr sites. A bulk crystal, which is not a phosphor but includes a small *W* local structure, can be a candidate for a narrow-band novel phosphor because crystals have similar local structures, suggesting a narrow-band emission. Examples include CaLiAl_3_N_4_[Ca], SrMg_3_SiN_4_[Sr], etc.

**Figure 6.**
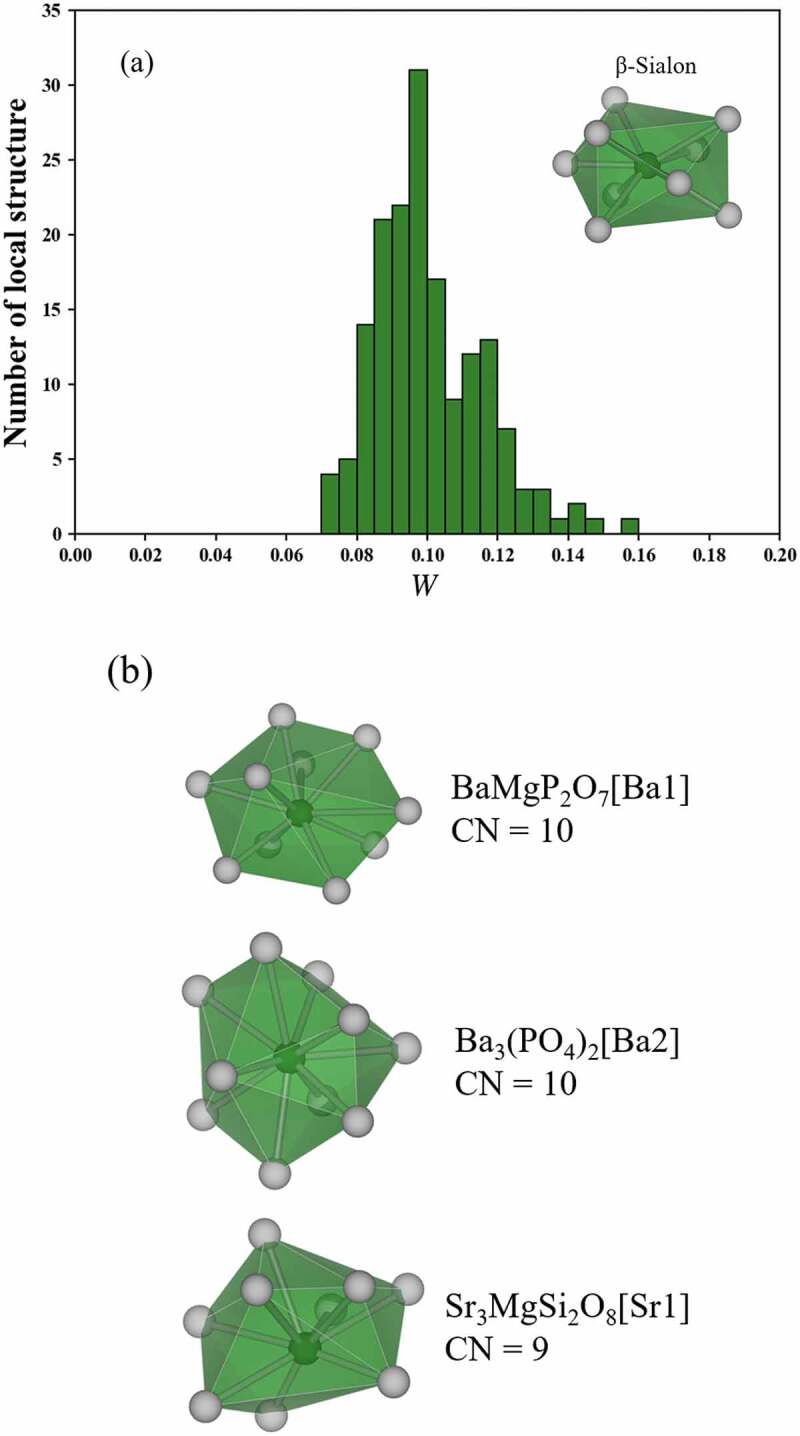
(a) Distribution of *W* to the 9-coordinated structure in β-Sialon and (b) the three local structures with the smallest *W* to the 9-coordinated structure in β-Sialon

Figure 6(a) shows the distribution of *W* to the 9-coordinated structure in β-Sialon. Since there is no local structure with *W* of 0–0.07, the local structures that selected in this study are dissimilar to 9-coordinated structure in β-Sialon. In order to confirm the dissimilarity, the three local structures with the smallest *W* to the 9-coordinated structure in β-Sialon are shown in Figure 6(b). BaMgP_2_O_7_[Ba1] and Ba_3_(PO_4_)_2_[Ba2] are dissimilar to 9-coordinated structure in β-Sialon because the coordination number of BaMgP_2_O_7_[Ba1] and Ba_3_(PO_4_)_2_[Ba2] is 10. The coordination number of Sr_3_MgSi_2_O_8_[Sr1] is 9 but the structure is a clearly dissimilar to 9-coordinated structure in β-Sialon. From these results, the 9-coordinated structure in β-Sialon are exceptional in Eu^2+^ phosphors. Therefore, if a crystal has a local structure with a very small *W* to the 9-coordinated structures in β-Sialon, the crystal is a promising candidate for a novel green phosphor with a narrow-band emission.

## 4. Conclusions

We proposed a (dis)similarity measure of the local structure using the Wasserstein distance to efficiently search for novel phosphors, and calculated the Wasserstein distance to obtain the quantitative dissimilarity of the local structures in crystals. Herein the local structures in crystals are numerically represented as a bag of the interatomic distances. The representation is invariant to the translation, rotation, and exchange of the order of ligands. This method is simple and versatile. It provides the quantitative dissimilarity for a local structure around the center ion.

The Wasserstein distance was calculated for various ideal and local structures in known phosphors. The variation of the Wasserstein distance corresponds to the structural variation of the local structures, and the Wasserstein distance can quantitatively explain the dissimilarity of local structures. Since *W* between the SrLiAl_3_N_4_[Sr1] and the SrLiAl_3_N_4_[Sr2] is 0.016, which is the minimum in this work, the two Sr sites are very similar. Therefore, the narrow-band emission of SrLiAl_3_N_4_:Eu^2+^ is due to the restraint in inhomogeneous broadening by the similarity of the two Sr sites.

A bulk crystal, which is not a phosphor but includes a small *W* local structure to SrLiAl_3_N_4_[Sr1], can be a candidate for a narrow-band novel phosphor because crystals with the local structures with the small *W* to SrLiAl_3_N_4_[Sr1], show a narrow-band emission. Examples include CaLiAl_3_N_4_[Ca], SrMg_3_SiN_4_[Sr] and RbLi(Li_3_SiO_4_)_2_[Rb]. On the other hand, there is no structure similar to the 9-coordinated structure in β-Sialon because *W* of the other local structures exceed 0.07. However, if a crystal has a local structure with a very small *W* to the 9-coordinated structure in β-Sialon, it will be a promising candidate for the novel green phosphor with a narrow-band emission.

The quantitative dissimilarity using the Wasserstein distance is useful in the search of novel phosphors and expected to be applied in materials searches in other fields in which local structures play an important role.
